# Year two of virtual interviews: longitudinal changes and diverse perspectives

**DOI:** 10.1186/s12909-023-04009-6

**Published:** 2023-01-19

**Authors:** Zachary Strumpf, Cailey Miller, Kaniza Zahra Abbas, Daniel Livingston, Ziad Shaman, Maroun Matta

**Affiliations:** 1 University Hospitals Cleveland Medical Center, Cleveland, Oh USA; 2 MetroHealth Medical Center, Cleveland, Oh USA; 3 Case Western Reserve University, Cleveland, Oh USA

**Keywords:** Virtual interview, Prospective applicants, Anonymous survey

## Abstract

**Background:**

The coronavirus disease (COVID-19) pandemic brought the virtual interview (VI) format to graduate medical education (GME) and the trainee recruitment process. It is unclear if applicants’ VI experience is consistent across all demographic groups. Our group collected 2 years of survey data to assess longitudinal changes in applicants’ attitudes towards the VI format. In addition, demographic data were collected, and analyses were performed to identify if between-group differences were present amongst a diverse applicant population.

**Methods:**

We distributed an anonymous electronic survey to applicants to the pulmonary disease and critical care medicine fellowship programs at Case Western Reserve University/University Hospitals Cleveland Medical Center and MetroHealth Medical Center for the 2021 and 2022 appointment years.

**Results:**

We received 112 responses (20% response rate) for our surveys. Although there was an overall stability of responses between the first 2 years, there were significant gender differences with applicants identifying as female more likely to recommend VI as a future model. Similarly, there were a significant difference in factor importance based on underrepresented minority (URM) status with applicants identifying as URM placing more emphasis on programs’ social media presence.

**Conclusions:**

There were no significant change in the responses of applicants between the first 2 years of VI. However, subset analyses revealed multiple significant findings. These differences have implications for future iterations of the VI format.

**Supplementary Information:**

The online version contains supplementary material available at 10.1186/s12909-023-04009-6.

## Background

Fellowship programs in pulmonary disease and critical care medicine (PCCM) have adopted the use of virtual interviews (VI) due to travel and safety recommendations resulting from the coronavirus disease (COVID-19) pandemic. The VI format was initially recommended by the Association of American Medical Colleges (AAMC) and the Alliance for Academic Internal Medicine (AAIM) for the 2020 recruitment season and the AAIM renewed this recommendation for the 2021 season as well [1, 2]. The VI experience, specifically as it relates to PCCM fellowship programs, has been previously studied [3, 4] yet questions of diversity and inclusion have remained and whether the apparent advantages (e.g., lower travel costs, reduced time off-service) and disadvantages (e.g., lack of meeting prospective faculty or co-fellows in person, placing interviewees at risk of not matching in their top programs) of a VI format were experienced equally among applicants of all backgrounds [3, 4]. This study aimed to utilize an online survey to evaluate if there were changes in attitudes towards the VI format between the first and second years of its implementation. In addition, we studied differences in those attitudes between various demographic groups of applicants.

## Methods

We distributed an anonymous online questionnaire to all applicants to the PCCM fellowship programs at either Case Western Reserve University/University Hospitals Cleveland Medical Center or Case Western Reserve University/MetroHealth Medical Center (584 total applicants) for the 2021 and 2022 appointment years. The 2022 questionnaire (included in the supplement) was identical to that described in our previous publication [3] with the addition of demographic data on each participant including: age, race/ethnicity, gender identity, medical degree held, visa status, and underrepresented in medicine (URM, as defined by the AAMC) status [5]. Invitations were sent on January 2, 2022, with up to two reminders sent every 5 days for those who had not yet completed the questionnaire. The questionnaires were converted to electronic format, and responses were anonymously collected and stored using REDCap electronic data capture tools hosted at Case Western Reserve University [6, 7]. A virtual interview satisfaction score for each applicant was created by assigning a Likert scale to the question “Do you believe virtual interviews will hurt or help your chances of getting into your top choices?” with “strongly hurt” assigned a score of one and “strongly helped” a score of five. Analysis for the collected responses included: mean with standard deviations used to describe participants characteristics, chi-squared test to evaluate for training amount and future model preference, a multinomial regression model was used to assess predictors of future interview preference and a linear regression model was used to assess predictors of applicants’ satisfaction with virtual interviews. All analysis was conducted using R version 4.1.0 [8]. The study was reviewed by the institutional review board (IRB) of Case Western Reserve University and received IRB exemption.

## Results

### Participant characteristics (Table 1- supplement)

One hundred and 12 (20%) participants completed the survey. Of the respondents, 62% identified as male and 38% as female. Forty-five respondents (43%) identified as underrepresented in medicine (URM).

### Longitudinal changes

**Fig. 1:**
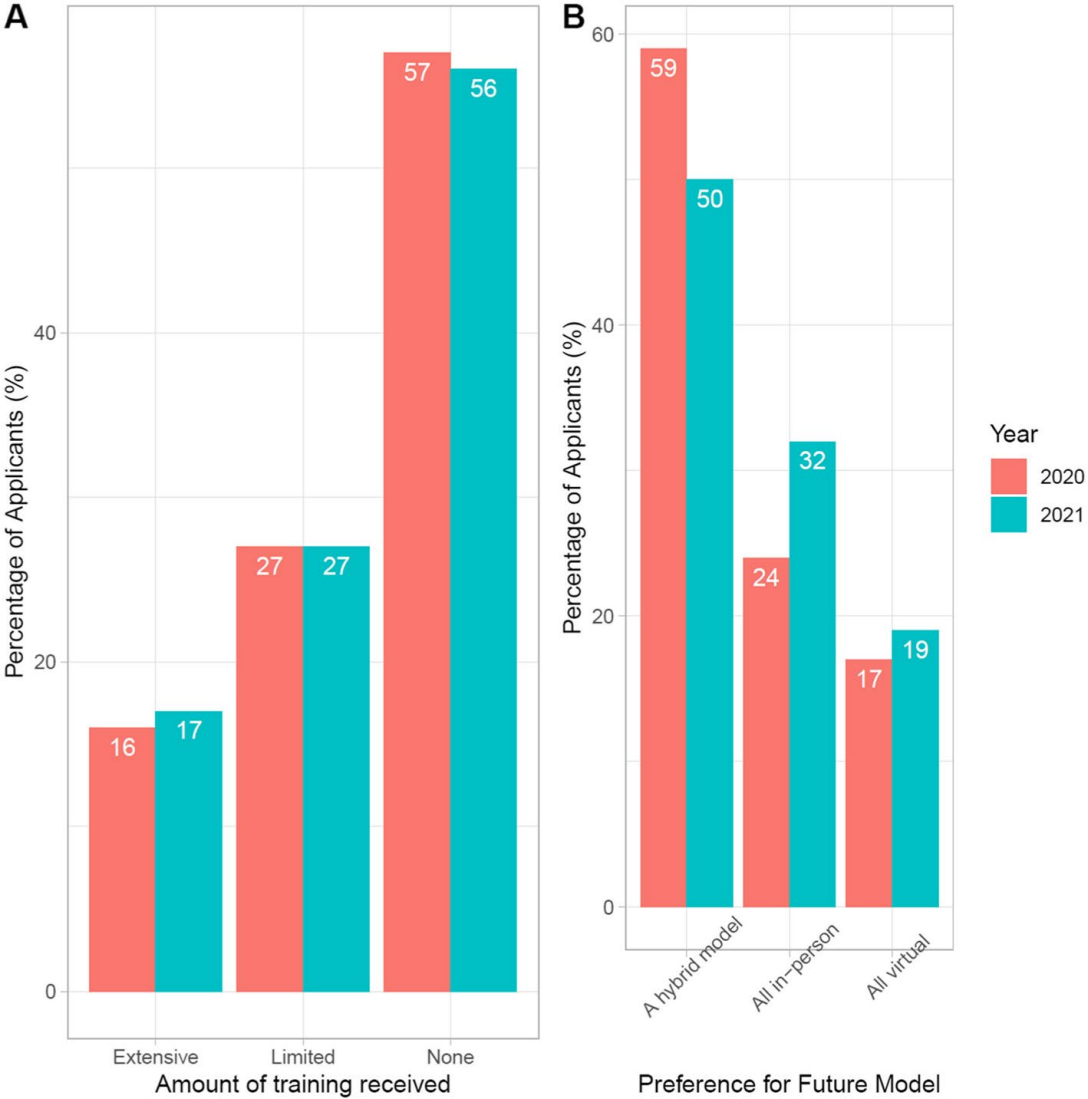
Change in amount of training received and preference for future interview model, 2020 versus 2021

There was no statistically significant difference in the reported amount of VI training applicants received (Pearson’s chi-squared test was performed with *p*-value of 0.98). There was also no statistically significant difference in applicants’ preference for interview model preference with the majority choosing a hybrid model both years (Pearson’s chi-squared test was performed with a *p*-value of 0.74) (Fig. 1).

### Group differences

A multinomial regression model evaluating applicants’ future interview preference as a function of age, gender, race, URM status, visa status and degree held, identified a significant difference in gender (*p* = 0.003), and J1 visa holders (non-United States citizens/immigrants working on a visa) (*p* = 0.02) preferring all-virtual interviews to all-in person.

**Fig. 2:**
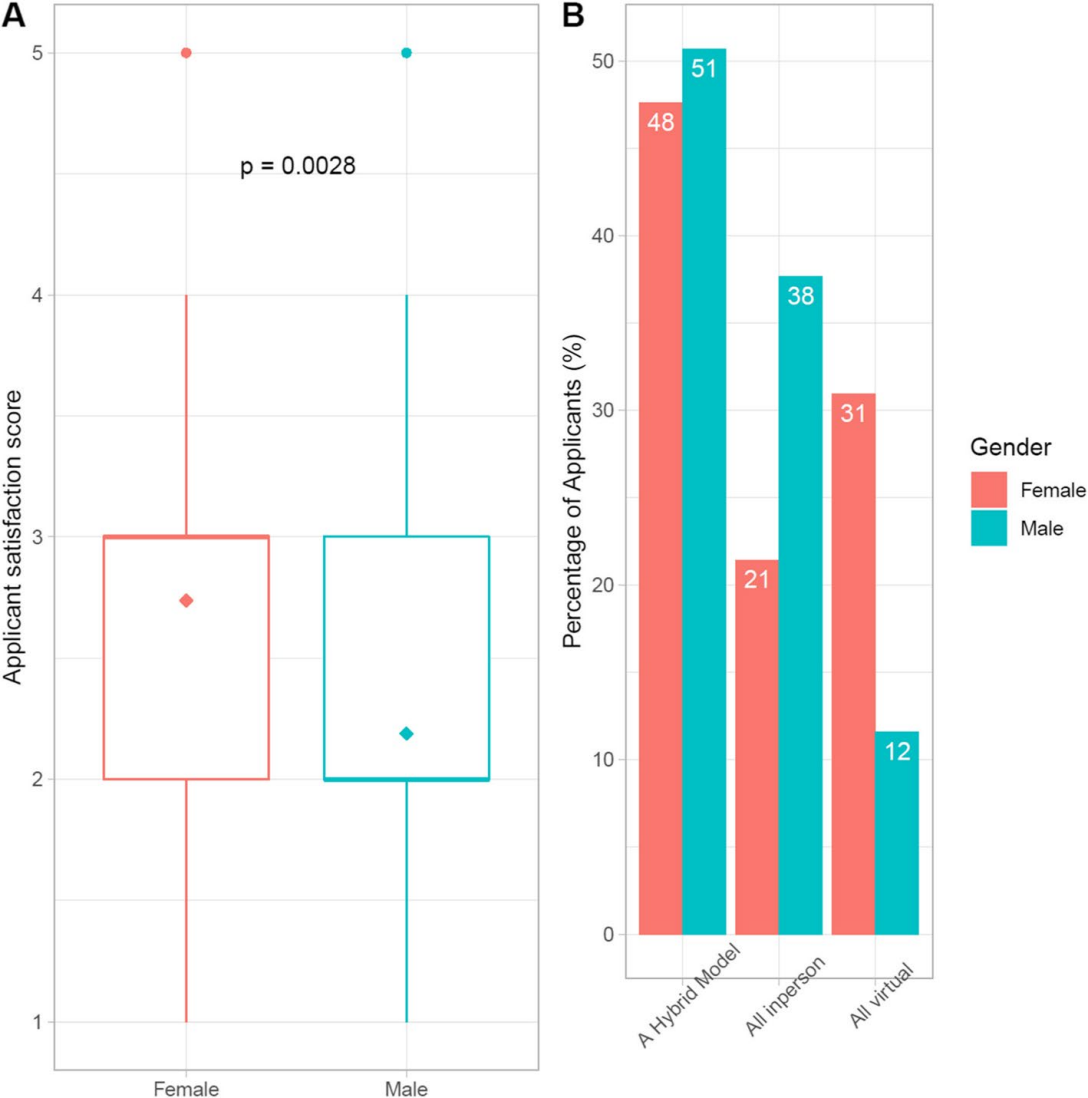
Applicant satisfaction score and preference for future interview model by gender

Participants who identified as female had a higher mean VI satisfaction score (2.74, SD = 1.01) compared to participants who identified as male (2.19, SD = 0.90) which was statistically significant (*p* = 0.003) (Fig. 2). The difference between “All in-person” (21% for participants who identified as female and 38% for participants who identified as male) and “All virtual” (31% for participants who identified as female and 12% for participants who identified as male) was statistically significant with *p*-value of 0.001.

Participants who identified as URM had a higher mean VI satisfaction score (2.41, SD = 0.9) compared to participants who did not (2.36, SD = 1.03) though the difference was not statistically significant (*p* = 0.67). There was no significant difference between VI model preference between those who identified as URM and those who did not.

In a preplanned subgroup analysis, we evaluated gender differences in importance of factors related to VI (Fig. S1 and S2 - Supplement). There were strong trends toward higher importance for participants who identified as male for doing a hospital tour (mean = 3.61, SD = 1.09) and visiting the city (mean 3.78, SD = 1) compared to participants who identified as female (mean = 3.2, SD = 1.09; mean 3.4, SD = 1.26, respectively), with *p*-values of 0.06 and 0.08 respectively. We also evaluated URM differences in importance of factors related to VI. There was a higher importance for programs’ social media presence (e.g., Instagram, Facebook, Twitter) in participants who identified as URM (mean = 2.86, SD 1.19) compared to participants who did not (mean = 2.33, SD 1.19) which was statistically significant (*p* = 0.02).

## Discussion

Our longitudinal data suggest that there was no change in the amount of training provided by residency programs for the VI format which may have left applicants unprepared for the VI format. Applicants’ overall preference for the three different interview models was also remarkably stable between the 2 years. The majority preference for a hybrid interview model, combining virtual and in-person components, remained the top choice for most of our participants. Although the attitudes of our participants towards the various factors of VI remained largely unchanged from last year, collection of demographic information revealed that there were many significant differences in the experiences of the VI format depending on the background of the applicant. Our data suggest that more women than men preferred an all-virtual interview experience. Our subset analysis of responses to the individual VI components suggests that factors like touring the hospital and visiting the city might be less important to women than men, although these differences did not reach statistical significance. Although URM status did not appear to influence the preference for a specific interview format, it was associated with a higher rating of the importance of programs’ social medial presence on decision making.

The limitations of our survey include overall low response rate and use of a regional sample of the national applicant pool. However, comparing our demographic data to that of the national applicant population did not reveal significant over- or under-representation of any demographic group. In addition, the timing of the survey is a potential confounder as it was performed post-match. Though this means that applicants were not biased to answer one way or another, it does mean that a varying period of time had elapsed from when the applicants had interviewed to when they had completed the survey. This also means that the applicant’s attitudes and responses might have been confounded given their perceived success or lack thereof during the interview season.

## Conclusions

Restrictions on travel and in-person meetings during the pandemic has provided a unique opportunity to evaluate the effect virtual interviews have had on training applicants’ experience.

Our results suggest that preparation of applicants is still lacking and that the experience of VI could vary widely for applicants of different backgrounds. To ensure equity in the recruitment process and the development of a diverse workforce in PCCM, future efforts should focus on clarifying the reasons for these demographic differences as well as strategies to address them.

## Supplementary Information

**Additional file 1:** **Table 1.** Participant demographics. **Supplementary Fig. S1.** Applicant satisfaction score and preference for future interview model by self-reported URM status. **Supplementary Fig. S2.** Importance factors for virtual interviews stratified by gender and self-reported URM status.

## Data Availability

Data and material is housed at University Hospitals, Cleveland Medical Center and might be available on a case-by-case basis for those who reach out. Please contact corresponding author Dr. Matta for queries regarding the data mentioned in this study.
