# Supplementary material for: Year two of virtual interviews: longitudinal changes and diverse perspectives
Source: BMC Med Educ. 2023 Jan 19;23:41. doi: 10.1186/s12909-023-04009-6 (PMC9850336; doi:10.1186/s12909-023-04009-6)
Supplement: Supplementary file 1 — Additional file 1: Table 1. Participant demographics. Supplementary Fig. S1. Applicant satisfaction score and preference for future interview model by self-reported URM status. Supplementary Fig. S2. Importance factors for virtual interviews stratified by gender and self-reported URM status. [file 12909_2023_4009_MOESM1_ESM.docx]

**Year Two of Virtual Interviews: Longitudinal Changes and Diverse Perspectives**

Zachary Strumpf, MD^1^, Cailey Miller, MD^1^, Kaniza Zahra Abbas, MD^1^, Daniel Livingston, MD^2^, Ziad Shaman, MD^2,3^, Maroun Matta, MD, MSc^1,3^

^1^University Hospitals Cleveland Medical Center, Cleveland, OH

^2^MetroHealth Medical Center, Cleveland, OH

^3^Case Western Reserve University, Cleveland, OH

Corresponding Author:

Maroun Matta, MD, MSc

Assistant Professor

Department of Medicine

Case Western Reserve University

Fellowship Director

Division of Pulmonary Critical Care and Sleep Medicine

University Hospitals Cleveland Medical Center

11100 Euclid Avenue, Bolwell

Cleveland, Ohio 44106

Email: maroun.matta@uhhospitals.org

**Online Supplement**


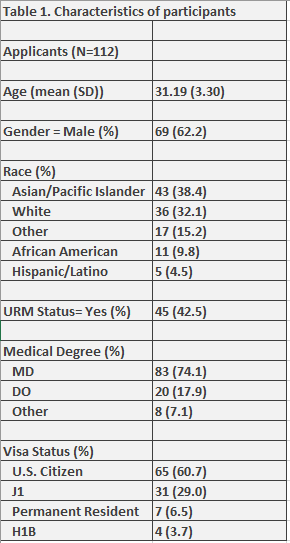


Table 1. Participant demographics.


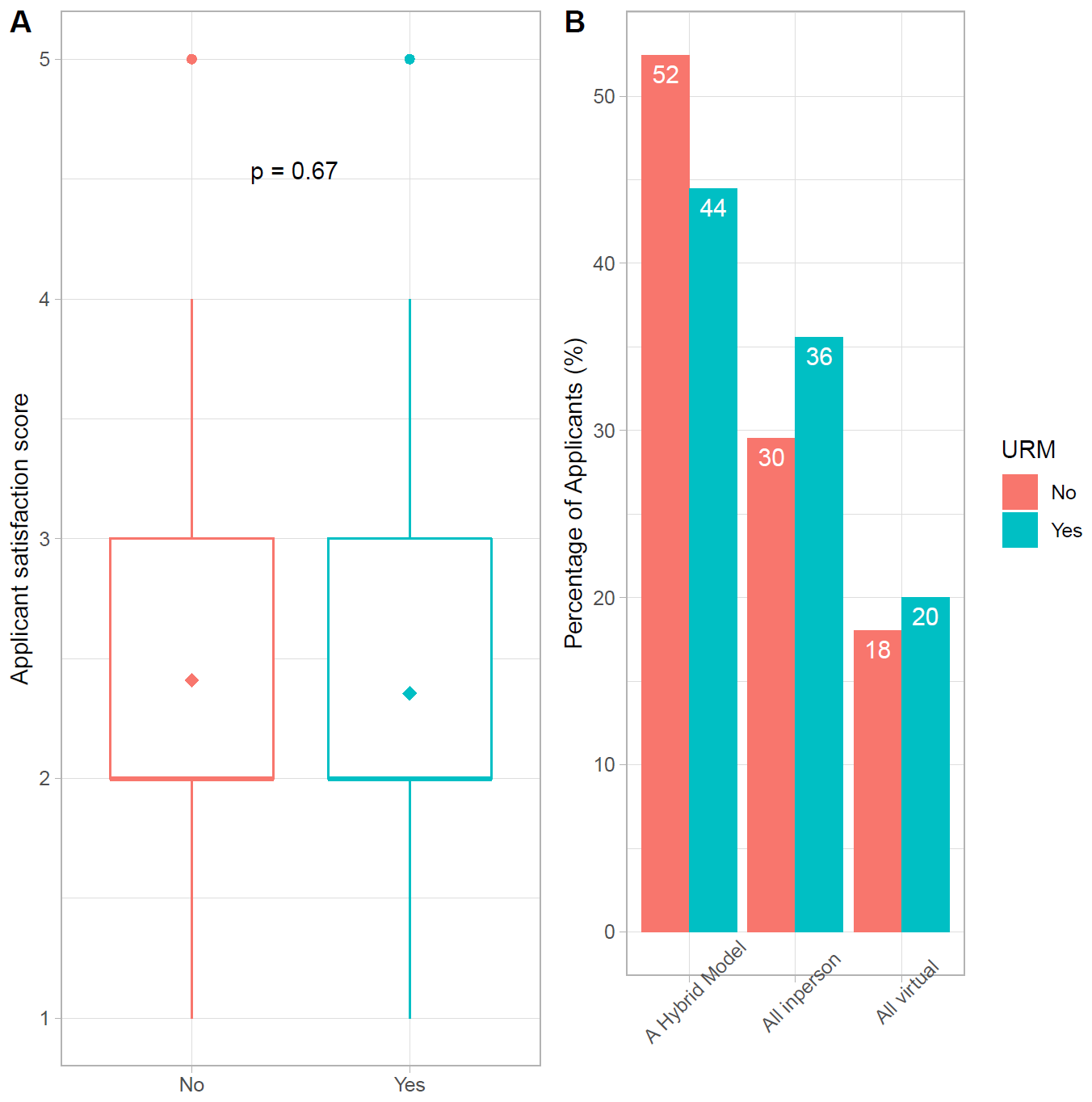


Supplementary Figure S1. Applicant satisfaction score and preference for future interview model by self-reported URM status.


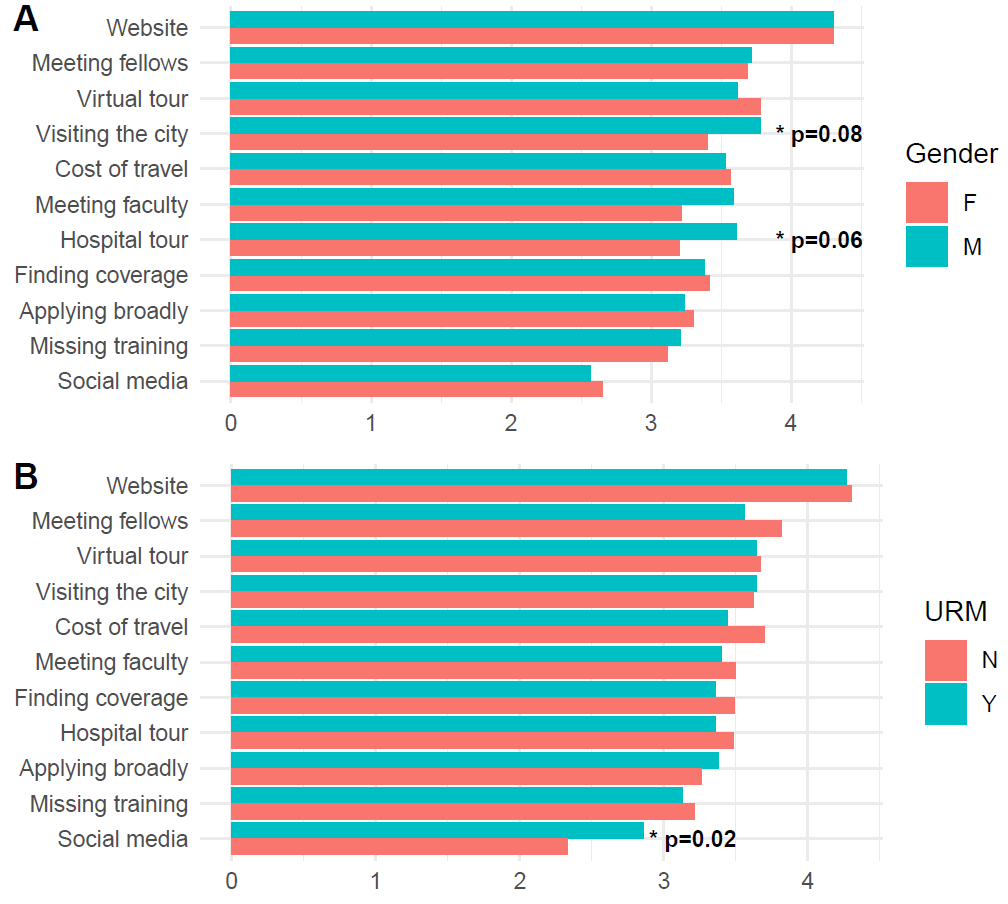


Supplementary Figure S2. Importance factors for virtual interviews stratified by gender and self-reported URM status.
